# Intensified plant N and C pool with more available nitrogen under experimental warming in an alpine meadow ecosystem

**DOI:** 10.1002/ece3.2583

**Published:** 2016-11-06

**Authors:** Fei Peng, Xian Xue, Quangang You, Manhou Xu, Xiang Chen, Jian Guo, Tao Wang

**Affiliations:** ^1^ key laboratory of desert and desertificationnorthwest institute of eco‐environment and resourceChinese Academy of Sciences; ^2^ University of Chinese Academy of SciencesBeijingChina; ^3^ Taiyuan Normal UniversityTaiyuanChina

**Keywords:** alpine meadow, incubation, infrared heater, microbial biomass, mineralization

## Abstract

Nitrogen (N) availability is projected to increase in a warming climate. But whether the more available N is immobilized by microbes (thus stimulates soil carbon (C) decomposition), or is absorbed by plants (thus intensifies C uptake) remains unknown in the alpine meadow ecosystem. Infrared heaters were used to simulate climate warming with a paired experimental design. Soil ammonification, nitrification, and net mineralization were obtained by in situ incubation in a permafrost region of the Qinghai‐Tibet Plateau (QTP). Available N significantly increased due to the stimulation of net nitrification and mineralization in 0–30 cm soil layer. Microbes immobilized N in the end of growing season in both warming and control plots. The magnitude of immobilized N was lower in the warming plots. The root N concentration significantly reduced, but root N pool intensified due to the significant increase in root biomass in the warming treatment. Our results suggest that a warming‐induced increase in biomass is the major N sink and will continue to stimulate plant growth until plant N saturation, which could sustain the positive warming effect on ecosystem productivity.

## 1 Introduction

Nitrogen (N) is considered to be the most important abiotic factor limiting net primary production in many terrestrial ecosystems like temperate and boreal forests, temperate grassland (Vitousek & Howarth, 1991), and the Arctic and alpine tundra (Chapin & Shaver, 1996; Jonasson, Michelsen, Schmidt, & Nielsen, 1999; Jonasson, Michelsen, Schmidt, Nielsen, & Callaghan, 1996; Zhang & Cao, 1999). Progressive N limitation may occur due to the coupling of carbon (C) and N in primary production if the warming‐induced increase in ecosystem C sequestration capacity is not accompanied by an increase in available N (Luo et al., 2004; Wu, Dijkstra, Koch, & Hungate, 2012). The positive effect of warming on C emission will partly be canceled out by intensified plant C sequestration capability (Contosta, Frey, & Cooper, 2011) if the N constraint on C uptake was alleviated (Natali, Schuur, & Rubin, 2012) with N mineralization stimulation under warming (Bai, Li, Li, Dai, & Jiang, 2013). Many studies have been conducted to investigate the effect of warming on N mineralization rate (Bai et al., 2013; Rustad et al., 2001), and high variable results were obtained because of the abiotic factor variations in different ecosystems (Bai et al., 2013; Brzostek et al., 2012). However, the elevated temperature is likely to precipitate the N cycling and SOM decomposition in the Arctic tundra (Jonasson, Michelsen, Schmidt, & Nielsen, 1999; Nadelhoffer, Giblin, Shaver, & Laundre, 1991) and alpine meadow ecosystems (Wang et al., 2012; Zhang et al., 2012).

The balance between mineralization and immobilization determines the available amount of inorganic N like ammonium (${\text{NH}}_{4}^{+}$) and nitrate (${\text{NO}}_{3}^{-}$) in soil, which are the main N sources available for plants. Microbial mineralization of soil organic matter (SOM) is low in tundra and alpine ecosystems due to the nutrient deficiency (Mack, Schuur, Bert‐Harte, & Shaver, 2004) and low temperature (Post, Emanuel, Zinke, & Stangenberger, 1982). Moreover, soil microbes compete with plants for the limited available N (Jonasson, Michelsen, & Schmidt, 1999; Kaye & Hart, 1997; Kuzyakov & Xu, 2013; Schmidt, Jonasson, Shaver, Michelsen, & Nordin, 2002). For example, a large amount of inorganic N is immobilized by microbes during the growing season (Hobbie & Chapin, 1998; Schmidt, Jonasson, & Michelsen, 1999), which results in N limitation for plant growth in tundra and the Arctic ecosystems (Clemmensen, Sorensen, Michelsen, Jonasson, & Ström, 2008; Schmidt et al., 1999; Sistla, Asao, & Schimel, 2012) yet increase in microbial decomposition of SOM (Jonasson, Michelsen, & Schmidt, 1999). While if plants outcompeted microbes for available N, ecosystem C uptake will be intensified (Jonasson, Michelsen, & Schmidt, 1999; Schmidt et al., 1999).

Permafrost soil in Qinghai‐Tibet Plateau (QTP) stores as much as 160 Pg C in the 0–25 m depth (Mu et al., 2015), equivalent to nearly 12% of global soil C storage in the top 1 m. It has a large potential for C emission under warming climate as permafrost C is very sensitive to global warming (Schuur et al., 2008). The net effect of warming on ecosystem C depends on the balance between elevated C emission through respiration and stimulated C uptake by photosynthesis. The C uptake and emission processes are all affected by the N availability under warming climate (Mack et al., 2004). We conducted a field experiment to examine the response of net mineralization rate, different N and C pools, and the coupled C and N responses to climate warming in an alpine meadow in the permafrost region of QTP.

## 2 Materials and Methods

### 2.1 Site description and experimental design

#### 2.1.1 Site description

**Figure 1:**
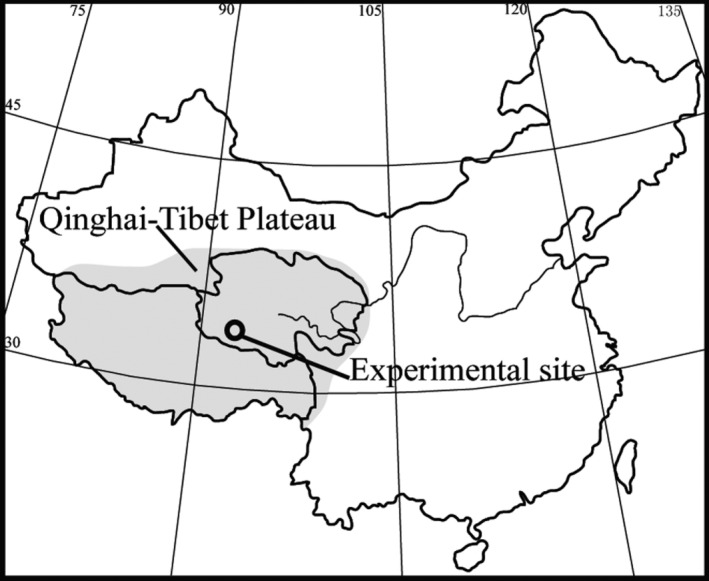
A map of the location of Qinghai‐Tibet Plateau and the study site

The study site is located in the headwater region of the Yangtze River and the middle of the QTP with a mean altitude of 4635 m and a typical alpine climate (Figure 1). Detailed information about the experimental site and soil features could be found in Peng, Xue, You, Zhou, and Wang (2015) and Table S1.

#### 2.1.2 Experimental design

A paired experimental design was used in this study. Ten 2 m × 2 m plots were divided into five pairs of control (unwarmed) and warmed plots. For each paired plots, the distance between the control (C) and the warmed (W) plot was at least 4 m to avoid the heating of the C plot by the infrared heater. The distance between each pair varied from 20 to 50 m. In each W plot, one 165 cm × 15 cm infrared heater (MR‐2420; Kalglo Electronics Inc., Utah, USA) was suspended in the middle at the height of 1.5 m above ground with a radiation output of 150 watts/m^2^. The heating began from July 1st, 2010 and continued over the entire study period. To simulate the shading effect of heaters, one “dummy” heater made of metal sheet with the same shape and size as the heater was installed in the C plots.

### 2.2 Measurement protocol

#### 2.2.1 Soil temperature and moisture

Thermistors were installed at soil depths of 5, 15, 30, 60, 100, 150, 200, 250, and 300 cm, respectively, to monitor the soil temperatures. EnviroSMART sensors based on frequency domain reflection were used to monitor volumetric soil moisture at 0–10, 10–20, 20–40, 40–60, and 60–100 cm depth. All the thermistors and soil moisture sensors were connected to a CR1000 data logger with a 10‐min interval. The 10‐min data were averaged into daily data when evaluating the warming effects on soil temperature and soil moisture.

#### 2.2.2 Ecosystem carbon fluxes

A shallow PVC collar (80 cm^2^ in area and 5 cm in height) was used for measuring soil respiration (Rs), and a deep PVC tube (80 cm^2^ in area and 50 cm in depth) was inserted into the soil in each plot near the shallow collar in July 2010 for measuring heterotrophic soil respiration (Rh). About 2–3 cm of either shallow or deep collars remained above the soil surface to facilitate field measurements. The deep PVC tube cuts off the old plant roots and prevents new roots from growing inside the tube. Carbon dioxide (CO_2_) efflux above deep tubes represents Rh after 3 months of deep collar insertion. Autotrophic respiration (Ra) was calculated as the difference between Rs and Rh (Zhou, Wan, & Luo, 2007). The measurement of Rs and Rh was conducted once or twice a month between 10:00 and 15:00 hr (local time) during the growing season, using a Li‐Cor 6400 portable photosynthesis system attached to soil CO_2_ flux chamber (6400‐09; Li‐Cor, Inc., Lincoln, NE, USA). Ecosystem respiration (ER) was measured with a transparent chamber (0.5 × 0.5 × 0.5 m) attached to an infrared gas analyzer (IRGA, Licor‐6400; Lincoln, NE, USA). The transparent chamber is a custom‐designed chamber made of Polytetrafluoroethylene (4 mm in thickness) with light transmittance about 99%. During measurements, a foam gasket was placed between the chamber and soil surface to minimize leaks. One small fan ran continuously to mix the air inside the chamber during measurements. Nine consecutive recordings of CO_2_ concentration were taken at a 10‐s interval during a 90‐s period in each measurement. The chamber was covered with an opaque cloth when measuring ER because the opaque cloth eliminates light (and hence photosynthesis). Carbon flux rates determined from the time‐course of the CO_2_ concentrations were used to calculate ER. The method used was similar to that reported by Steduto, Çetinkökü, Albrizio, and Kanber (2002) and Niu et al. (2008).

#### 2.2.3 Biomass

Aboveground biomass (AGB) was obtained from a stepwise linear regression with AGB as the dependent variable, and coverage and plant height as independent variables (AGB = 22.76 × plant height + 308.26 × coverage − 121.80, *R* ^2^ = .74, *p* < .01, *n* = 100, Xu et al., 2015). Coverage of each experimental plot was measured eight times using a 10 cm × 10 cm frame in four diagonally divided subplots. Plant height was measured 40 times by a ruler and averaged for each experimental plot. Root biomass (RB) was sampled using a soil corer with the internal diameter of 7 cm. One core was extracted every 10 cm between 0 and 50 cm depth in the center of each experimental plot during the growing season (from May to September) 2012, providing five soil layers (0–10, 10–20, 20–30, 30–40, and 40–50 cm). After root and soil samples were collected, they were immediately placed in a cooler and transported to the laboratory by train. In the laboratory, soil samples were air‐dried and crumbled by hand to pass through a 2‐mm‐diameter sieve in order to remove large particles from the finer soil. Subsequently, living fine roots were hand‐picked based on their color and consistency in a distilled water bath (Yang, Fang, Ji, & Han, 2009). The remaining soil (without roots) was used to measure soil inorganic N. The separated fine roots were dried at 75°C for 48 h and weighed.

#### 2.2.4 Carbon and nitrogen concentration and C and N pools of plant

Concentrations of total C and N of root biomass in May and September were analyzed in an elemental analyzer (Elementar Vario Macro cube, Hanau, Germany), and N pool of root biomass was obtained by multiplying RB and its N concentration.

#### 2.2.5 Soil inorganic nitrogen concentration and pool

Soil ammonia (${\text{NH}}_{4}^{+}$) concentration was measured using the Nessler reagent colorimetric method. The Nessler reagent was made up of HgI_2_‐KI‐NaOH. Approximately 5 g of soil sample was extracted in a centrifuge tube with 25 ml of 2 mol/L KCl, oscillated for about 30 min, and filtered through filter paper to get the supernatant. Before measuring on the spectrophotometer at the wavelength of 490 nm, sodium tartrate about 25%, acacia about 1%, and Nessler reagent were added into the supernatant (Lu, 2000). Soil nitrate (${\text{NO}}_{3}^{-}$) concentration was measured using the Phenoldisulfonic acid colorimetric method. About 5 g of soil was put into a centrifuge tube, and 25 ml of CuSO_4_ of 0.02 mol/L was added into the soil, and then, the mixture was oscillated for about 10 min and filtered. The ${\text{NO}}_{3}^{-}$ was measured at the wavelength of 420 nm on a spectrophotometer. Before measuring on a spectrophotometer, MgCO_3_ and phenol disulfonic acid were added into the extract (Lu, 2000). Total soil inorganic N in each layer was the sum of ${\text{NH}}_{4}^{+}$ and ${\text{NO}}_{3}^{-}$.

#### 2.2.6 Mineralization

Mineralization was measured by an in situ incubation technique (Eno, 1960). It consists of measuring differences between the initial and final inorganic N content in closed containers placed in the soil, which assumed micro‐environmental conditions inside the container are similar to those in the surrounding soil (Mazzarino, Oliva, Abril, & Acosta, 1991). The technique prevents plant uptake of mineralized nutrients but allows uptake by the microbial biomass (Schmidt et al., 1999). In our practice, a PVC collar (80 cm in area and 50 cm in depth) was inserted to each plot, and the top of each collar was covered with Parafilm, which could prevent the input of wet deposition of N to the collar by rainfall. Soil moisture in each plot was modulated near to the field capacity on May 11, 2012 (28% cm^3^/cm^3^). Soil samples were collected at the depth of 0–10, 10–20, and 20–30 on May 11th, 2012 and September 2nd, 2012. The ${\text{NH}}_{4}^{+}$ and ${\text{NO}}_{3}^{-}$ concentrations were measured using the same approach as for measuring the soil inorganic N. Ammonification and nitrification were calculated as the difference of ${\text{NH}}_{4}^{+}$ and ${\text{NO}}_{3}^{-}$ of soil samples between September 2nd and May 11th, 2012. Net mineralization was the sum of ammonification and nitrification.

#### 2.2.7 Microbial carbon and nitrogen

Microbial carbon (MBC) and nitrogen (MBN) were measured using the fumigation–extraction technique (Li, Wang, Yang, Gao, & Liu, 2011). About 5 g of soil was extracted with 0.5 M K_2_SO_4_ for an hour. Another set of 5 g of soil fumigated with ethanol‐free chloroform for 24 h was extracted in the same manner. Both the nonfumigated and fumigated soil extracts were filtered through Whatman GF/D filter. The difference between the concentrations in the fumigated and nonfumigated extracts was used to derive MBC and MBN. Soil samples were collected at 0–10 depth in May and September 2012.

### 2.3 Data analysis

The warming experiment began from July 2010. Meteorological and soil microclimate data from July 2010 to the end of 2012 were used in the analysis. *Repeated measurement analysi*s that best solve the autocorrelation of measurements (Karvchenko & Robertson, 2015; Webster & Payne, 2002) was used to test the warming effect and its interaction with measuring month and with soil depth as a covariate on soil ammonia, nitrate, total inorganic nitrogen, and root biomass. Two‐way ANOVA analysis was used to test the effects of warming and measuring month on MBC, MBN, N concentration of roots, and aboveground biomass. Seasonal ammonification, nitrification, net mineralization, microbial immobilization, and soil inorganic N pool change were calculated as the difference of inorganic or microbial N in each layer between September and May. One‐way ANCOVA was used to test the warming effect on ammonification, nitrification, and mineralization with soil depth as a covariate. When analyzing the warming effect on ammonification, nitrification, and mineralization in each soil depth, *paired T*‐test was used. Before doing the test, normality of variables and equal variance between groups was tested. The variable will be log‐transformed if variables did not meet normality requirement. Soil inorganic N pool was obtained by multiplying inorganic N concentration and soil bulk density in the corresponding layer, and N pool in 0–50 cm was the sum of all layers. Plant N pool was attained by multiplying root N concentration and RB. *Paired T*‐test was employed to examine the warming effects on microbial N pool, soil inorganic N pool, and root N pool.

## 3 Results

### 3.1 Microclimate

Higher mean annual temperature and annual precipitation were recorded in 2012 (−3.5°C and 420.1 mm) in comparison with the long‐term average (−5.1°C ± 0.13 and 294.5 ± 23 mm 1981–2008). Soil temperature significantly increased by 2.27°C (*p *< .01, Figure S1) at the depth of 5 cm, and soil moisture declined by 1.12% (cm^3^/cm^3^, *p *< .01) in 0–10 cm soil layer in W plots. The positive warming effect on soil temperature could reach to 2 m, and the warming impact on soil moisture turned from negative to positive at 40 cm depth.

### 3.2 Ecosystem carbon fluxes and biomass

**Figure 2:**
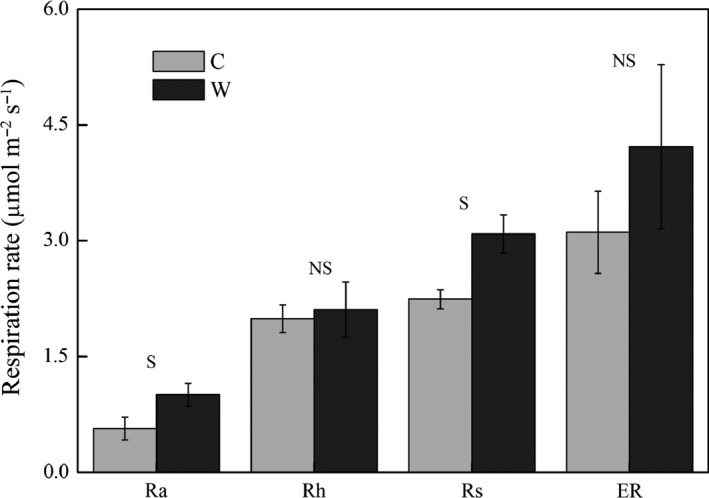
The seasonal average of soil respiration (Rs) and its components (Ra, autotrophic respiration, and Rh, heterotrophic respiration), and ecosystem respiration (ER) under control (C) and warming (W) treatments. NS and S represent significant or nonsignificance difference at *p *< .05 level

**Figure 3:**
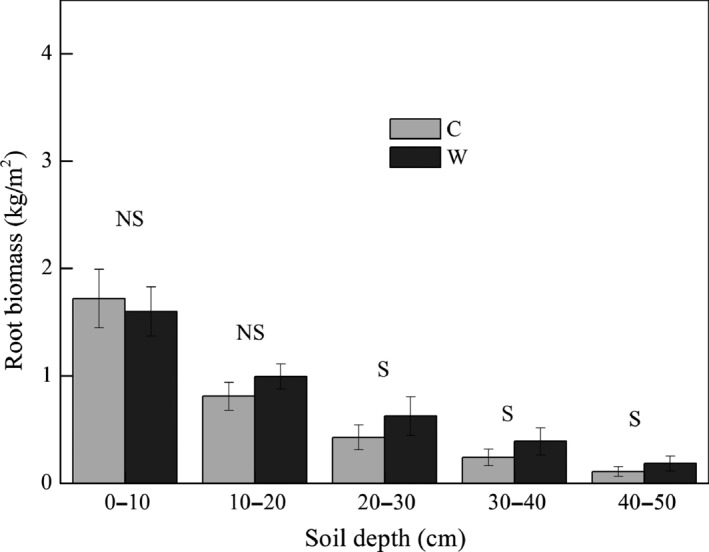
The seasonal average of root biomass in different soil layers (0–10, 10–20, 20–30, 30–40, 40–50 cm) and in the total 0–50 soil profile under control (C) and warming (W) treatments. NS and S represent significant or nonsignificance difference at *p *< .05 level

Average Rs and Ra were significantly increased by 77% and 38% (*p *< .05), while Rh and ER had no significant (*p *> .05) change in warming plots (Figure 2). Warming had no significant effect on AGB (Table 1, *p* > .05), but it significantly increased RB by 0.48 kg/m^2^ (*p *< .05) when RB in 0–50 cm summed together (Figure 3). Interaction of warming with measuring month had no significant effect on AGB and RB (Table 1, *p* > .05).

**Table 1:** Results (*F*‐values) of repeated measurement analysis with measuring month (*M*), warming treatment (*W*) as factors and soil depth (*D*) as a covariate on concentration of soil ammonia (${\text{NH}}_{4}^{+}$), nitrate (${\text{NO}}_{3}^{-}$), total inorganic N and root biomass (RB); results of two‐way ANOVA analysis with measuring month and warming treatment as factors on microbial biomass (MB), nitrogen (MN), N concentration of roots (NC_RB), and aboveground biomass (AGB); results of one‐way ANOVA with warming treatment as factor and soil depth as a covariate on ammonification, nitrification, and mineralization

	*M*	*W*	*D*	*M* × *W*	*M* × *D*
${\text{NH}}_{4}^{+}$	6.5**	0.1	29.3**	0.9	0.8
${\text{NO}}_{3}^{-}$	14.0**	0.2	1.4	0.3	0.2
Inorganic N	10.53**	0	6.1**	0.5	0.7
RB	24.3**	0.9	110.0**	0.9	19.3*
MB	3.6*	0		0.9	
MN	1.6	0.2		0.9	
NC_RB	1.5	0.9		0.8	
AGB	25.8**	1.5		0.3	
Ammonification		1.6	4.6*		
Nitrification		3.5^	13.3**		
Mineralization		4.7*	5.1*		

Significance: ^*p* < .1; *p < .05; **p < .01.

### 3.3 Net ammonification, nitrification, and mineralization

**Figure 4:**
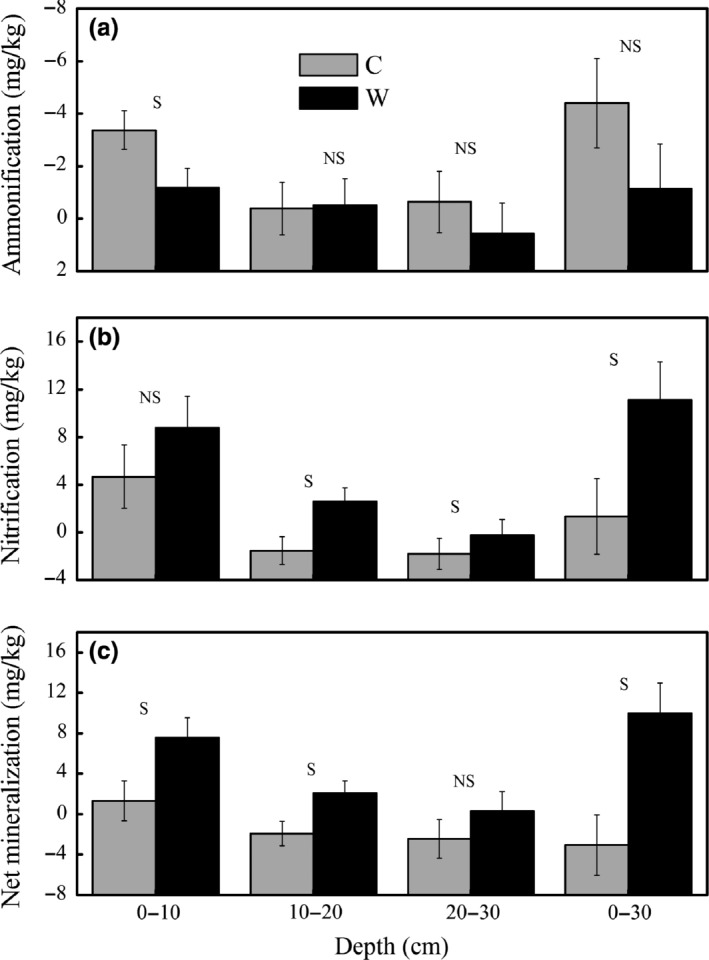
Soil ammonification, nitrification, and mineralization potential under control (C) and warming (W) in different soil layers. The ammonification, nitrification, and net mineralization were the difference of concentrations of them between September and May. NS and S represent significant or nonsignificance difference at *p *< .05 level

Ammonification, nitrification, and net mineralization rate changed significantly among 0–10, 10–20, and 20–30 cm soil layers (Figure 4, *p* < .05). Warming marginally increased nitrification rate (*p* = .078) and significantly increased net mineralization rate (Table 1, Figure 3, *p* = .047). Net mineralization positively correlated with nitrification rate (*r* = .95, *n* = 6) thus increased nitrification resulted in mineralization rate increase under warming.

### 3.4 Microbial C and N

**Figure 5:**
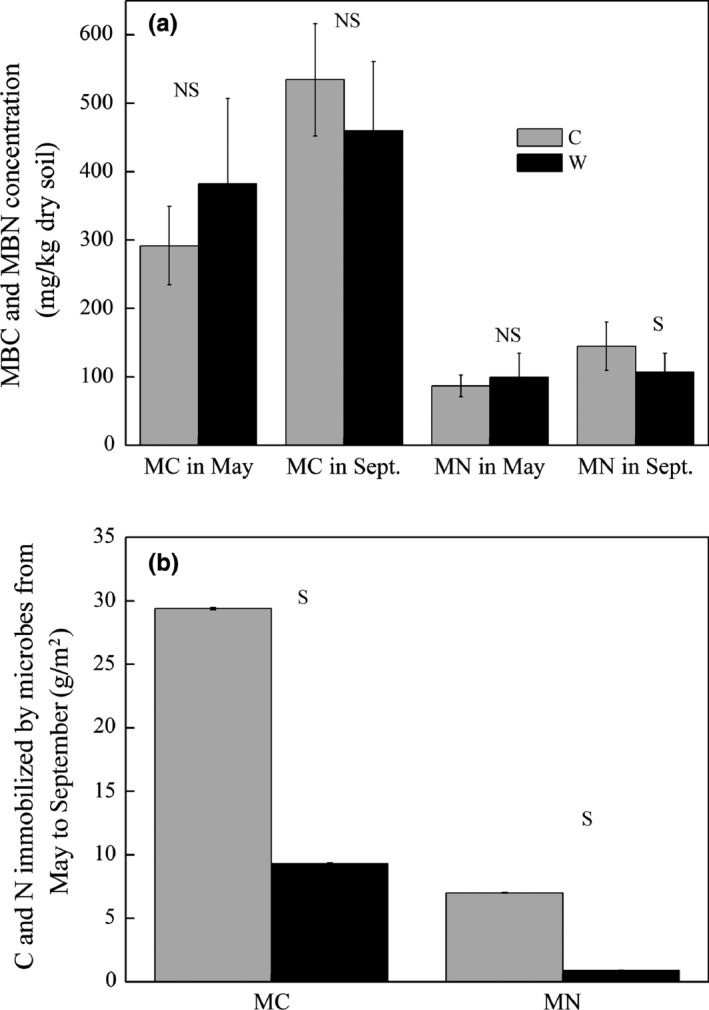
Microbial carbon (MBC) and nitrogen (MBN) in May (dark gray columns) and September (black columns), 2012 under control (C) and warming (W) treatments (a), and delta MBC and MBN between the 2 months under C and W (b). S in (a) represents significant differences of MBC or MBN between C and W in May or September, and in (b) represents significant difference of the MBC or MBN change between September and May in C and W

Warming, measuring month, and their interaction had no significant effect on MBC and MBN (Table 1, *p* > .05). Microbes immobilized C and N from May to September (Figure 5). Warming significantly decreased the magnitude of immobilized C and N in microbes (Figure 5b, *p* < .05). The microbial C: N marginally increased (*p* = .05) in W plots (3.7 vs. 4.2 in C and W plots, respectively).

### 3.5 Soil inorganic N

**Figure 6:**
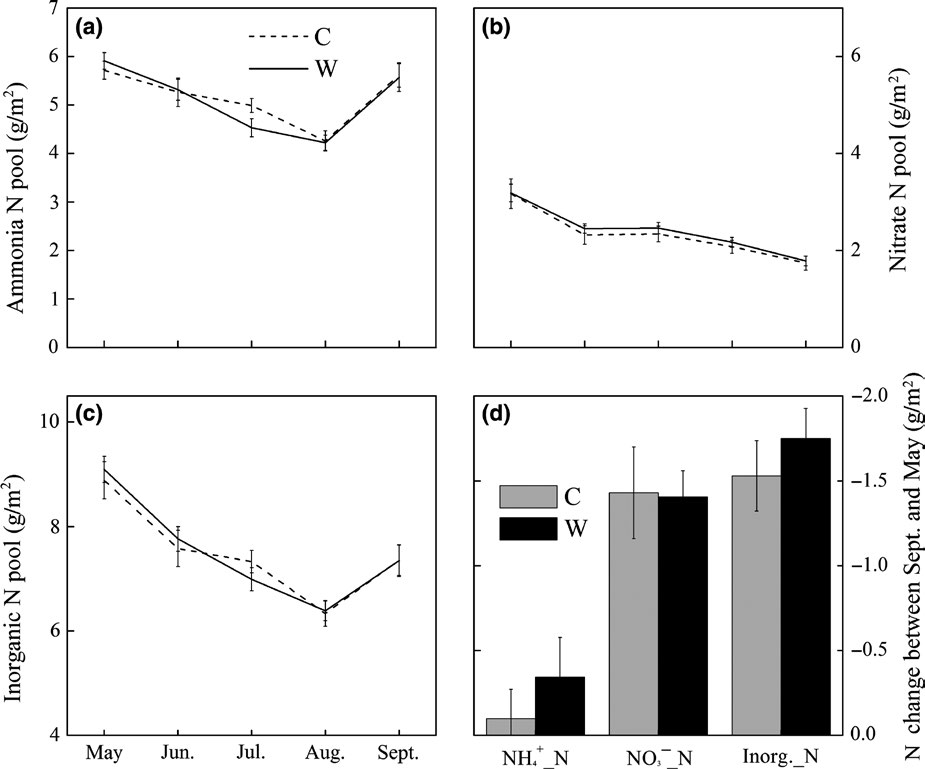
Soil ammonia (${\text{NH}}_{4}^{+}$, a), nitrate (${\text{NO}}_{3}^{-}$, b), and inorganic N pool (c) in 0–50 cm soil profile in different months from May to September, and the soil N pools change (d) between September and May in C and W treatments

Soil ${\text{NH}}_{4}^{+}$, ${\text{NO}}_{3}^{-}$, and the sum of ${\text{NH}}_{4}^{+}$ and ${\text{NO}}_{3}^{-}$ showed seasonal variations, which were higher in the beginning of the growing season, began to decrease with plant growth and finally recovered at the senescence of plants to the initial values in the beginning of the growing season (Figure 6a–c). Soil lost ${\text{NH}}_{4}^{+}$, ${\text{NO}}_{3}^{-}$, and total inorganic N during a growing season (negative values, Figure 5d). Warming had no significant effect on the magnitudes of soil ${\text{NH}}_{4}^{+}$, ${\text{NO}}_{3}^{-}$, and soil inorganic N loss (Figure 6d). Soil C:N was about 11, and warming had no significant effect (*p *> .05) on it.

### 3.6 Root N concentration and pool

**Figure 7:**
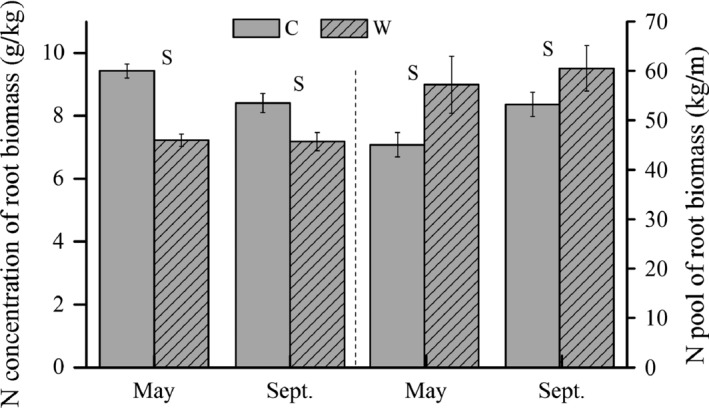
Roots N concentration and pools in May and September under C and W treatments. N concentration of root biomass is the average of five layers (0–10, 10–20, 20–30, 30–40, and 40–50), and root N pool was the total storage in 0–50 cm

Warming significantly decreased average N concentration (*p *< .05) of roots in 0–50 cm by 19% but enhanced the N pool by 20% (Table 1, Figure 7). Average C:N ratio was significantly higher in W (57.9 ± 2.3) than in C plots (48.3 ± 2.9). The total N absorbed by roots in 0–50 cm was 15.3 ± 3.8 and 16.8 ± 2.3 g/m^2^ in C and W, respectively, in a growing season.

## 4 Discussion

### 4.1 Warming effects on N mineralization, ammonification, nitrification, and plant and soil N pool

Response of net N mineralization to warming is highly variable among ecosystems (Bai et al., 2013; Rustad et al., 2001) with different mean annual temperatures or incubation temperatures (Nadelhoffer et al., 1991), soil moistures (Emmett et al., 2004), and duration of the warming manipulation (Wang et al., 2014). The net mineralization rate would increase when the mean annual temperature is about 9–15°C (Nadelhoffer et al., 1991). Although the mean annual temperature is a minus in our study site, relatively high average soil temperature during the growing season in 0–5 cm (8.8 and 10.6°C in C and W, respectively) might result in the increase in N mineralization (Figure 4). Moreover, the stimulated SOM decomposition, especially in rhizosphere indicated by the significant increase in Rs and Ra (Figure 2, *p* < .05), may also contribute to the increase in organic N mineralization. The presence of plants will stimulate N mineralization presumably due to more release of N in the rhizosphere (Jonasson, Castro, & Michelsen, 2006). The significant increase in root biomass (Figure 3, *p* < .05) therefore probably could result in the increment in N mineralization, which, in turn, stimulate the plant productivity because N limitation for plant growth is alleviated (Shaver & Chapin, 1995).

The net N mineralization and nitrification show a positive correlation (Bai et al., 2013; Owen, Wang, Wang, King, & Sun, 2003); thus, the net mineralization change could explain the increase in nitrification in the current study. Warmer climate favors nitrifying microbes with an optimum temperature of 20°C in subarctic regions (Dalias, Anderson, Bottner, & Coûteaux, 2002). Nitrification increase should be expected if warming brings the soil temperature closer to the optimum in cold environments. Soil temperature in growing season in the current study is only 8.8°C, but the warming‐induced increase in soil temperature drives it closer to 20°C and therefore could probably be one reason for the nitrification increase. Nitrification response to warming is also affected by soil moisture (Bai et al., 2013). The highest increase in nitrification appears in an ecosystem where soil moisture is moderate (55% water‐filled pore space, Szukics et al., 2010). Although reduced, the moderate mean growing season soil moisture (57% water‐filled pore space) could support the positive response of nitrification to warming.

Warming may reduce labile C for microbes and has a negative effect on microbial biomass (Bradford et al., 2008), whereas increased N availability could stimulate microbial growth (Yin, Chen, & Liu, 2012). Provided more available N, outcompetition of plants comparing with microbes for the inorganic N (Dong, Scagel, Cheng, Fuchigami, & Rygiewicz, 2001; Schmidt et al., 2002) could explain the reduction in microbial immobilization of C and N (Figure 5b). Plant N is mainly stored in the live roots, which could account for 79.6% of the plant N (Zhang & Cao, 1999). Although N pool of AGB has not been examined, the enhanced root N pool (Figure 7) with a reduction in MBC and MBN (Figure 5b) indicates that plants use the increased available N in a warming climate.

Ammonia‐oxidizing bacteria (AOB) could impact the seasonal dynamic of soil ${\text{NH}}_{4}^{+}$ and ${\text{NO}}_{3}^{-}$ as AOB is positively related to potential nitrification rate. The seasonal change of AOB community positively correlated with soil temperature and pH (Zhang, Qin, Ren, Li, & Yang, 2008). Therefore, the decrease in soil ${\text{NH}}_{4}^{+}$ and inorganic N from May to August and recovery in September (Figures 5c and 6a) could be the result of the seasonal dynamic of AOB driven by soil temperature. Sustained reduction in soil ${\text{NO}}_{3}^{-}$ (Figure 6c) from May to September corresponds to the plant phenology as alpine plants show a preference for ${\text{NO}}_{3}^{-}$ (Xu et al., 2011).

### 4.2 Interactions between plants and microbes in cycling of nitrogen

**Figure 8:**
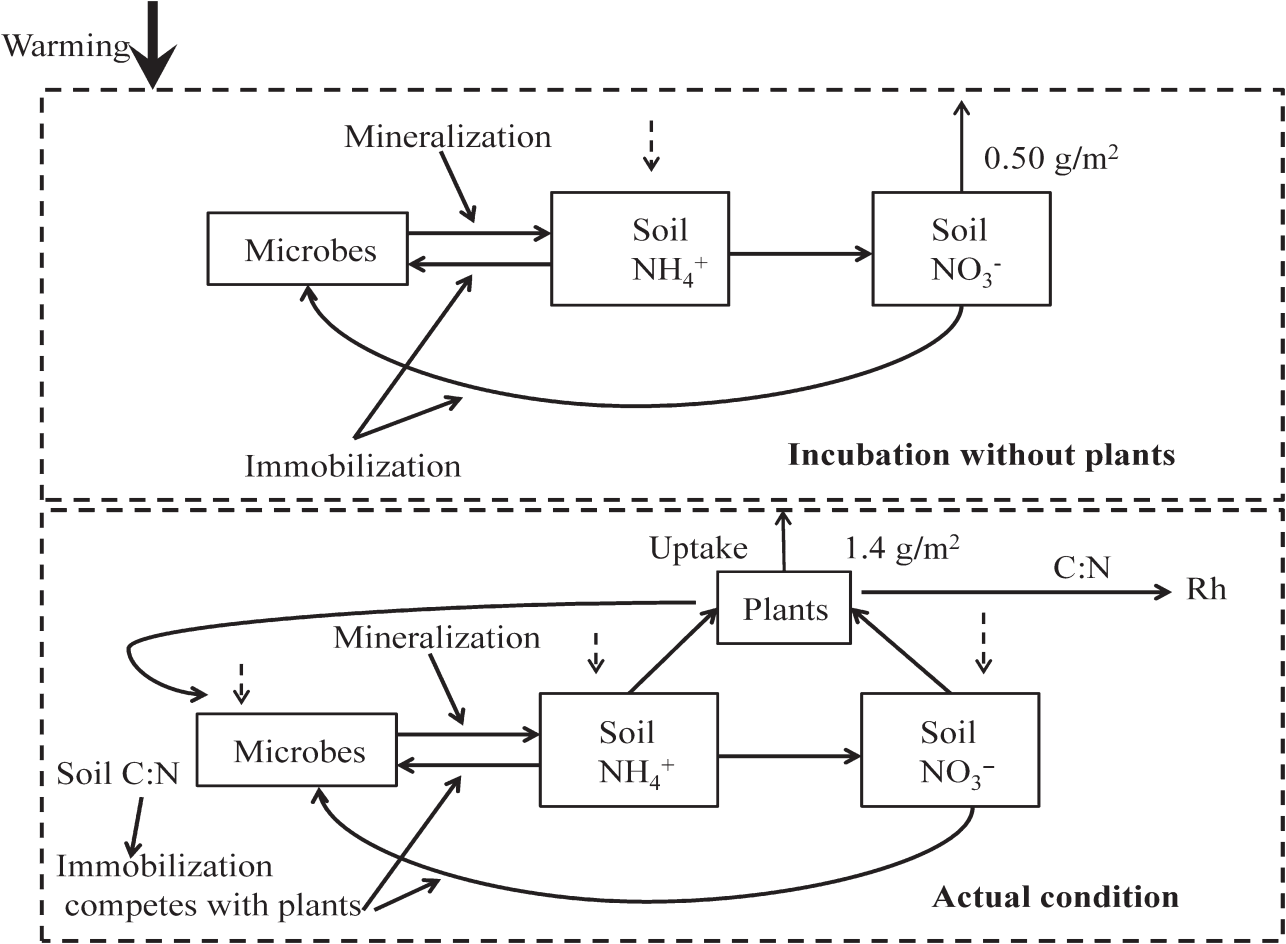
Change in ecosystem N compartments with increased mineralization rate with and without plants under a warming climate. ↑means increase and ↓means decrease, solid lines mean significant change, and dash lines mean nonsignificant change

The significant increase in available soil inorganic N and plant N pool with warming could probably be caused by enhanced N supply (Dijkstra et al., 2010). The increased mineralization rate in the current study (Figure 4) supports the expected substantial increase in N supply with moderate increment in soil temperature in a short run (Kuzyakov & Xu, 2013). The temporal niche differentiation of microbes and plants is highly relevant ecologically because it could continuously provide roots with available N according to plant demand and contribute to the evolutionary development of mutualism interactions between roots and microorganism (Kuzyakov & Xu, 2013). Roots provide microorganisms with C, and in turn, roots obtain nutrients because microorganisms efficiently decompose nutrients from sources that are chemically or spatially unavailable to plants (Kuzyakov & Xu, 2013). Even a small fluctuation in the microbial N content will increase or decrease plant available nutrients to an extent of potentially affecting plant growth (Schmidt et al., 2002). If the turnover of microbial biomass and microbial N is much shorter than growing season length, the microbial N could have been recycled several times. At the same time, plants obtain multiple opportunities to sequester the recycled N, which could result in high sequestration and retention of N in the plants over the growing season (Schmidt et al., 2002), but would not show up as increased N pools in the microbial biomass (Hodge, Robinson, & Fitter, 2000). Reduction in microbial immobilization of N (Figure 5b) and nonsignificant change in soil ${\text{NO}}_{3}^{-}$ (Figure 5), but increased nitrification rate (Figure 4b) and intensified root N pool (Figure 7) implies that more available N mainly flows to the plant N pool under warming (Figure 8), which is similar to the study in the Arctic ecosystems (Jonasson et al., 2006).

### 4.3 Coupled response of carbon and nitrogen to warming

Plant organs with high C to N ratio are less digestible and slow the rates of litter decomposition (Welker, Fahnestock, Sullivan, & Chimner, 2005). The reduction in root N concentration therefore could lead to the nonsignificant change in Rh (Figure 1) and suggests that warming has no significant effect on bulk soil SOM decomposition. N is intimately linked to C cycling as both decompositions of soil organic C by microbes, and the photosynthetic uptake of atmospheric CO_2_ by plants is limited by N availability. The increase in net mineralization and Rs in the current study support the positive effect of elevated temperature on gross N mineralization and organic matter decomposition (Feng et al., 2007). However, the nonsignificant increase in Rh without the presence of plants and the significant increase in Ra (Figure 2) indicate a warming‐induced increase in Rs mainly derives from the stimulation of decomposition of root exudates as increased N supply stimulates plants productivity in terms of increase in root biomass (Figure 3). Increases in mineralization and intensified plant N pool indicate plant uptake is the main N sink. Plant C stimulation will sustain until the plant N saturated according to the original N saturation framework (Niu et al., 2016), while further long‐term studies should be conducted to certify the coupling responses of N and C processes to climate warming.

## 5 Conclusion

In the alpine meadow ecosystem in the permafrost region of QTP, warming significantly increased nitrification and net N mineralization, consequently elevated the N supply. Plant N pool in roots intensified even though the N concentration of roots decreased because of the significant enhancement in RB. The Ra especially the Rs increase resulted from increased RB, but a nonsignificant change in Rh without the presence of plants suggests that the enhanced ecosystem C emission might primarily the result of stimulation of SOM decomposition in the rhizosphere. Direct positive effects of warming on C emission will be partly offset by the direct stimulation of plant growth and indirectly constrained by a reduction in bulk SOM decomposition due to increased C to N ratio of root litter in the alpine meadow in the QTP.

## Conflict of Interest

None declared.
