# Supplementary material for: Intensified plant N and C pool with more available nitrogen under experimental warming in an alpine meadow ecosystem
Source: Ecol Evol. 2016 Nov 6;6(23):8546–55. doi: 10.1002/ece3.2583 (PMC5167058; doi:10.1002/ece3.2583)
Supplement: Supplementary file 1 [file ECE3-6-8546-s001.docx]

Supplement materials

Table S1 Soil properties of the control plots

| Soil depth (cm) | TOC | LC | CaCO3 | TN | IN | pH |
| --- | --- | --- | --- | --- | --- | --- |
| 0-5 | 8.96±3.21 | 3.01±1.66 | 5.98±0.75 | 0.51±0.20 | 4.02±2.01 | 8.31±0.10 |
| 5-10 | 8.35±2.63 | 3.14±1.48 | 6.21±0.91 | 0.47±0.15 | 3.35±1.47 | 8.39±0.10 |
| 10-20 | 7.33±3.02 | 3.23±1.55 | 6.22±0.91 | 0.39±0.15 | 3.12±1.62 | 8.43±0.06 |
| 20-30 | 6.04±2.06 | 3.08±1.85 | 6.51±0.90 | 0.34±0.12 | 2.33±0.92 | 8.47±0.06 |
| 30-50 | 6.24±2.46 | 3.26±1.99 | 6.41±1.05 | 0.35±0.14 | 3.08±2.36 | 8.50±0.08 |

SOC is the soil organic carbon (g kg-1), LC is the labile carbon (g kg-1), TN is the total nitrogen (g kg-1), CaCO3 represents the inorganic carbon (g kg-1), IN is the sum of N-NO3- and NH4+ (mg kg-1)

Fig. S1 Seasonal dynamic of daily rainfall, soil temperature at 0-5cm depth and soil moisture at 0-10 layer in control (C) and warming (W)
